# Coupling Two Different Nucleic Acid Circuits in an Enzyme-Free Amplifier

**DOI:** 10.3390/molecules171113211

**Published:** 2012-11-06

**Authors:** Yu Jiang, Bingling Li, Xi Chen, Andrew D. Ellington

**Affiliations:** Center for Systems and Synthetic Biology, Department of Chemistry and Biochemistry, Institute for Cellular and Molecular Biology, University of Texas at Austin, Austin, TX 78712, USA

**Keywords:** catalyzed hairpin assembly (CHA), hybridization chain reaction (HCR), 96-well plate, enzyme-free

## Abstract

DNA circuits have proven to be useful amplifiers for diagnostic applications, in part because of their modularity and programmability. In order to determine whether different circuits could be modularly stacked, we used a catalytic hairpin assembly (CHA) circuit to initiate a hybridization chain reaction (HCR) circuit. In response to an input nucleic acid sequence, the CHA reaction accumulates immobilized duplexes and HCR elongates these duplexes. With fluorescein as a reporter each of these processes yielded 10-fold signal amplification in a convenient 96-well format. The modular circuit connections also allowed the output reporter to be readily modified to a G-quadruplex-DNAzyme that yielded a fluorescent signal.

## 1. Introduction

Enzyme-free DNA circuits [1] that rely only on hybridization and strand-exchange reactions to achieve signal amplification have seen increasing use as diagnostic assays. Two of the more robust and programmable DNA circuits are catalytic hairpin assembly (CHA) [2] and the hybridization chain reaction (HCR) [3,4]. CHA and HCR have, respectively, proven useful for amplifying and transducing signals from nucleic acid and other analytes into fluorescent, electrochemical, or colorimetric readouts [2,5,6,7], while HCR has also been used for multiplexed imaging of endogenous mRNA in fixed zebra fish embryos [8].

CHA has proven to be particularly good at staged amplification, while HCR is particularly good at amplification on surfaces such as electrodes or magnetic beads [3,9,10,11]. Therefore, we hypothesized that a heterogeneous platform that started with CHA amplification and subsequent immobilization of the amplification products might be further amplified by HCR. We were further encouraged by the fact that basic methodologies for heterogeneous platforms (*i.e.*, ELISA assays) have proven extremely successful; for example, the 96-well Neutr-avidin plate used herein has already been widely proofed in assay development [12,13].

**Scheme 1:**
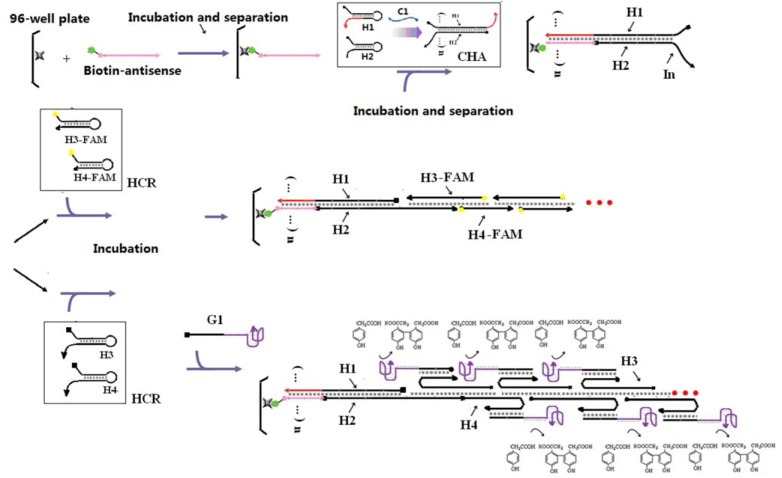
Scheme for CHA-HCR two-layer DNA amplifier.

In our conception (Scheme 1), a single-stranded nucleic acid analyte(C1) can initiate the CHA reaction. The accumulated duplex products [n(H1:H2)] have exposed single-stranded DNA tails at the 3' ends of both H1 and H2. The H1 tail can be further immobilized by hybridization to a Biotin-antisense oligonucleotide on a plate. Following washing, this amplified surrogate of the original nucleic acid analyte provides a substrate for HCR, which can conveniently initiate via the H2 tail (In). The two HCR substrates (H3 and H4) assemble into long concatameric chains [In:(H3:H4)n], and an output signal can be directly read based on the fluorescent labels placed at the 3’ends of H3 and H4. 

The coupled reaction in fact showed coupled amplification, indicating the relative simplicity of ganging together nucleic acid circuits for diagnostic applications. In order to further demonstrate the versatility of the reaction, we transduced the initial signal into a fluorescent reporter, a G-quantet-hemin DNAzyme.

## 2. Results and Discussion

### 2.1. Designing a CHA-HCR Circuit

Catalytic hairpin assembly (CHA) and the hybridization chain reaction (HCR) are nucleic acid circuits in which kinetically trapped conformers can react in the presence of a single-stranded sequence-specific trigger or catalyst to form new, more energetically stable conformers. While each reaction starts from two hairpin substrates, CHA yields many duplexes, while HCR yields a double-stranded concatamer.

**Scheme 2:**
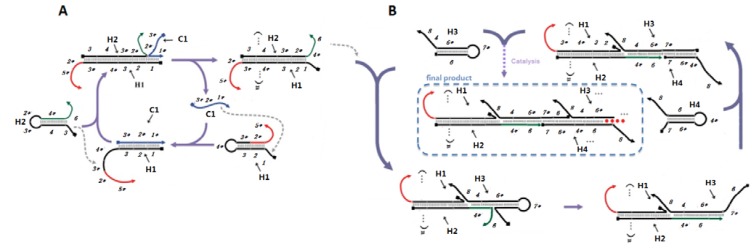
Detailed scheme of CHA (**A**) and HCR (**B**) systems.

In order to combine these two circuits, such that CHA would trigger HCR, we first had to position the single-stranded catalyst for HCR on the CHA product (Scheme 2A). The CHA circuit that we used was based on a circuit that had previously shown low background and good amplification [2]. As shown in Scheme 2A, domain 1* (eight residues) on C1 binds to the toehold domain 1 on the first hairpin substrate (H1), initiating a fast branch migration [14] reaction that presents domain 3*-4* in a single-stranded conformation. This domain then triggers a second strand displacement with H2.

The sequences of the CHA substrates were modified so that the free 3’ end tails on H1 (2*-5*) and H2 (4*-6) could participate in other hybridization and/or strand-exchange reactions. The 16-residue tail on H1 allows hybridization of the double-stranded CHA product to an antisense sequence to the 16-residue tail (Biotin-antisense) modified streptavidin-coated plates. Since domain 2* is hidden in the initial H1 hairpin, the substrates should not efficiently hybridize to the plates. Single-stranded domain 4*-6 will initiate the HCR. 

The HCR circuit was designed based on the principles set out in [4]. The two complementary hairpin substrates (H3, H4) both contain 18 base-pair stems, six residue loops, and six residue toeholds (Scheme 2B). Upon interaction with domain 4*-6 of an immobilized H1 with the complementary 4-6* domain on H3, a strand displacement reaction will lead to opening of H3 and release of the 7*-6 domain, which can in turn hybridize the 7-6* domain on H4, thereby initiating another strand displacement reaction. The newly freed 4*-6 domain on H4 can in turn open a second H3, followed by iterative opening and reaction of H4, H3, and so forth. As a result of HCR H3 and H4 should join to form an elongated assembly product (as shown in Scheme 2B). Note that compared to the 8-base toehold for the CHA circuit, the HCR toehold had only six residues. Therefore, in order to improve the rate of reaction initiation, we used higher salt concentrations (0.66 M NaCl) and lower temperatures (25 °C) in the HCR portion of the coupled reaction.

The ultimate formation of the HCR product was monitored by including fluorescein at the 3' ends of H3 and H4. However, the coupled CHA-HCR cascade should be comparable with a wide range of reporters [10,15,16,17]. To prove this point, we further replaced the FAM tag on H3 and H4 with polyT_18_ tails that could hybridize to a polyA_18_-G-quartet-hemin complex. The G-quartet-hemin complex is a well-known horseradish peroxidase-like DNAzyme that can catalyze the oxidation of p-hydroxyphenylacetic acid (HPA) to the fluorescent product bi-p,p-4-hydroxyphenylacetic acids (Bi-HPA) in the presence of H_2_O_2_. The measured emission at 410 nm should reflect the presence and concentration of the catalyst strand C1.

### 2.2. Characterization of the CHA and HCR Circuits

**Figure 1:**
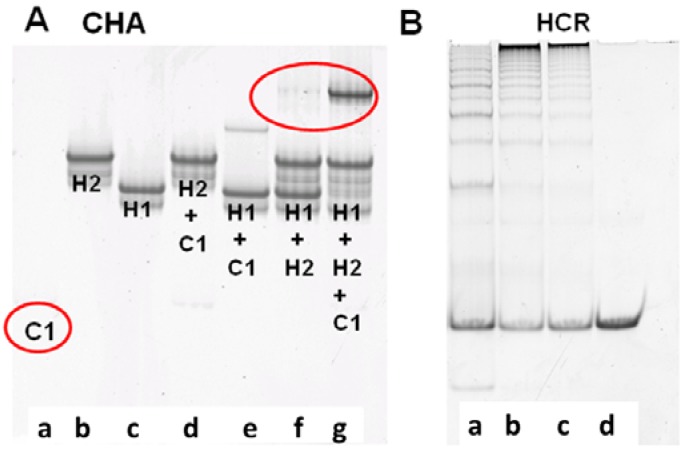
Verification of CHA and HCR products. (**A**) 15% PAGE gel after 3-hour CHA reaction. Concentrations of C1, H1, and H2 (in a to g) are 10 nM, 200 nM, and 400 nM, respectively; (**B**) 8% PAGE gel after overnight HCR reaction. Concentrations of H3 and H4 are both 200 nM. Concentrations of the initiator mimic (InM) from **a** to **d** are 100 nM, 50 nM, 25 nM, and 0 nM.

The performance of CHA and HCR circuits was separately verified by native polyacrylamide gel electrophoresis (PAGE), which could reveal what products were being formed as the reaction progressed. For the CHA reaction, after 3 h of incubation with 10 nM C1, 200 nM H1, and 400 nM H2, a single high molecular weight product corresponding to H1:H2 appears, while the band corresponding to H1 almost completely disappears [Figure 1A(g))]. While some background duplex formation could be observed even in the absence of C1 [Figure 1A(f)] the rate of leakage is slow and is likely due to either mis-synthesis and/or mis-folding of H1 and H2.

The performance of the CHA circuit could also be confirmed by including an additional substrate, which upon interaction with H1:H2 would lead to strand displacement and release of a FAM-labeled oligonucleotide from a quencher-labeled antisense molecule. As shown in Figure S1, both the C1-dependent catalytic reaction and the background leakage were consistent with the PAGE results (Figure 1A). Since the balance between adequate signal and minimal noise could be most readily observed after a 3 h reaction, a 3 h pre-CHA reaction was chosen to initiate the HCR reaction. 

To independently proof the HCR reaction, an initiator mimic (InM, representing the single-stranded 4*-6 sequence) was used to initiate the reaction. Figure 1B shows the native gel profile following overnight incubation of H3 and H4 with different concentrations of InM. It is obvious that long duplex concatamers (InM:(H3:H4)_n_) are produced in the presence of the InM. Unsurprisingly, the greater the amount of InM the shorter the concatamers produced, since the same amount of substrate is being simultaneously added to multiple, growing HCR chains. The HCR substrates were almost consumed within 5 h (Figure S1), and the fluorescence of the coupled CHA-HCR cascade could therefore generally be readily monitored after an overnight reaction.

### 2.3. Two-Layer Amplification by CHA-HCR

**Figure 2:**
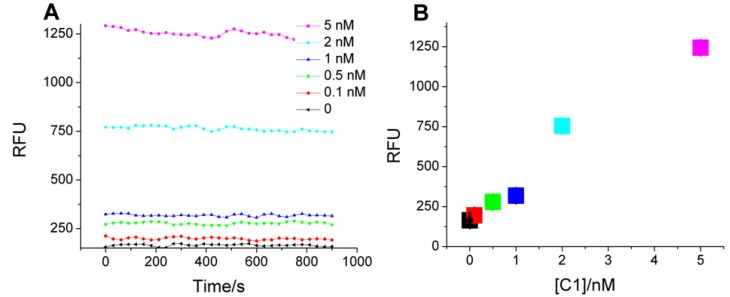
Kinetics of two-layer amplification by CHA-HCR. (**A**) Fluorescent kinetic curves after standard CHA-HCR two-layer amplfication; (**B**) Concentration dependence of C1 following standard CHA-HCR two-layer amplfication. The RFU for each concentration is the mean of all steady-state data points in Figure 2A.

For assaying analytes, the coupled system was transferred onto microtiter plates. As in Scheme 1, catalyst-dependent CHA in the first stage should produce a sequence-specific bridge to fluorescent HCR products in the second stage. Samples with C1 indeed produce much higher fluorescence compared with the blank (no C1; Figure 2A), and the mean fluorescence intensity (calculated from the data points at each C1 concentration) is concentration-dependent (Figure 2B), with a linear correlation coefficient of 0.94. As little as 100 pM C1 (three standard deviations above background) could be discriminated from the blank, a limit similar to “on-plate” or “on-fiber” sensors using protein enzymes or nanoparticles for signal amplification [10,15,16,17]. 

**Figure 3:**
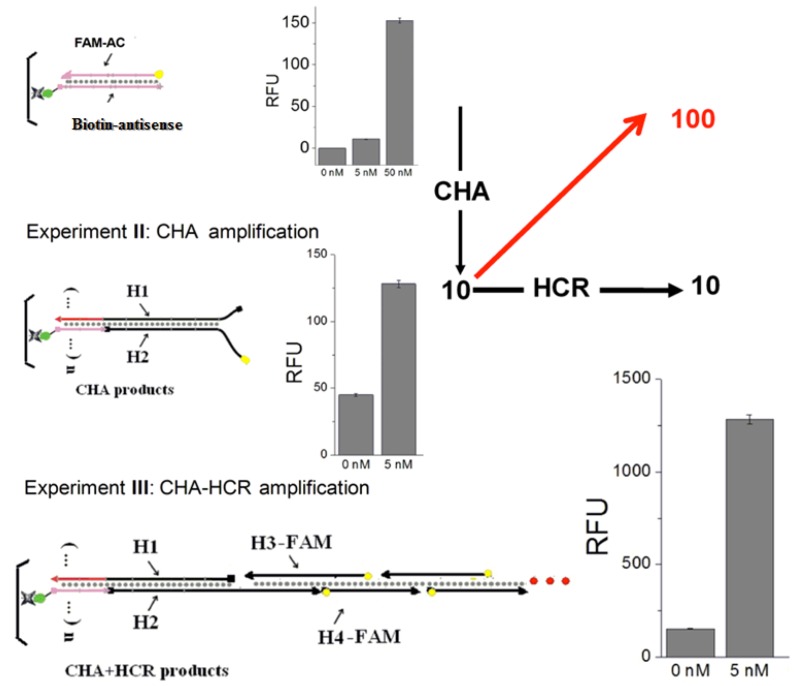
Confirmation of the two-layer amplifier. Experiment I:Without any amplification. Experiment II: With only CHA amplifier. Experiment III: With the standard CHA-HCR amplifier. The RFU in each bar graph is the mean of the steady-state data points collected during fluorescence kinetic readings.

To confirm that both CHA and HCR were contributing to analytical sensitivity, two control experiments are carried out (Figure 3). First, a FAM-labeled complement (FAM-AC) to the immobilized Biotin-antisense was hybridized to mimic a simple DNA sensor without any amplification. Second, FAM-labeled H2 (FAM-H2) was included in the CHA circuit. After triggering by C1, the CHA products (H1:H2 (FAM)) was hybridized with the Biotin-antisense on plates, to mimic a system with CHA but without HCR amplification. An analyte-dependent fluorescence increase could be observed in both experiments. However, the signal aptitude and sensitivity were much less than the standard CHA-HCR amplification (Figure 3, Experiment III). Based on the data in Figure 5, and Figure 6A, it can be calculated that the signal resulting from 5 nM analyte was 10-fold increased by CHA, and additionally 10-fold increased by HCR, for an overall amplification of ca. 100-fold, confirming two-layer amplification. 

Even greater amplification was limited by the unexpectedly high blank signal (no C1). This signal likely results from CHA leakage, HCR leakage, and non-specific binding to the plate. 

### 2.4. G-quartet-hemin DNAzyme as a Reporter

As well known, some G-quartets can bind hemin and thereby form a peroxidase DNAzyme [18,19] G-quartet-hemin DNAzymes catalyze a series of colorimetric and luminous oxidization reactions in the presence of H_2_O_2_, and have been widely used as protein-free signal amplifiers and reporters in biosensors [18,19]. We therefore decided to test whether the DNAzyme might provide additional amplification in our coupled CHA-RCA assay. A fluorescent peroxidase substrate (HPA) was chosen and its turnover was found to be dependent upon both pH and the sequence of the G-quartet. The G-quartets ‘G1’ and a pH of 8.5 were chosen for sensing reactions (Figure S2). 

**Figure 4:**
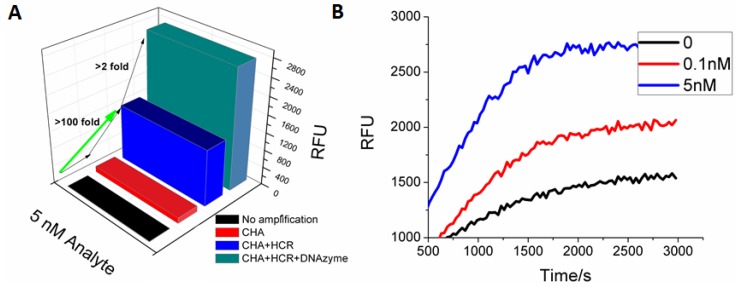
G-quartet-hemin DNAzyme as a reporter for CHA-HCR. (**A**) Steady-state signal comparison between no amplification, CHA amplification, standard CHA-HCR two-layer amplification, and CHA-HCR-DNAzyme amplification. The RFU is the mean of the steady-state data points collected during fluorescence kinetic readings; (**B**) Fluorescent kinetic measurement with CHA-HCR-DNAzyme amplification.

In order to utilize the DNAzyme with the HCR product, the FAM tags on H3 and H4 were replaced with polyT_18_ tails that could hybridize to a polyA_18_-G1-hemin DANzyme (Scheme 1). Following the coupled reaction, a C1-dependent fluorescent signal could be observed in the presence of HPA (Figure 4). At the previous limit of detection (100 pM), the DNAzyme gave a 10-fold higher signal amplitude than the FAM reporter. At higher (saturating) concentrations of C1 (5 nM) the DNAzyme still gave a 2-fold higher fluorescent response relative to the FAM reporter. As before, accumulation of background limits sensitivity and signal. It should be noted that though as little as 5 pM of analyte could be reproducibly detected (three standard deviations above background), the DNAzyme reporter did not show a linear dose-response curve (Figure 4B, Figure S3), again in part a consequence of the aforementioned background.

## 3. Experimental

### 3.1. Chemicals and Oligonucleotides

All chemicals were of analytical grade and were purchased from Sigma-Aldrich (St. Louis, MO, USA) unless otherwise indicated. All oligonucleotides were ordered from Integrated DNA Technologies (IDT, Coralville, IA, USA). Oligonucleotide sequences are summarized in Supplementary Table S1. All DNA hairpins (including H1, H2, H3, and H4) were purified via denaturing PAGE (7 M Urea, 1×TBE). All DNAs were stored in 1×TE (pH 7.5) at a concentration of 1 mM. Pierce NeutrAvidin Coated High Capacity Plates (Black, 96-Well) were purchased from Thermo Scientific (Waltham, MA, USA). The wash buffer for the plate was Tris-buffered saline (25 mM Tris, 150 mM NaCl; pH 7.2), 0.1% BSA, and 0.05% Tween®-20 detergent.

### 3.2. CHA Reactions on the Plate Surface

Each well was washed three times with 200 μL of wash buffer. Some 100 μL of 1 μM Biotin-antisense in 1×TNaK (20 mM Tris-HCl, 140 mM NaCl, 5 mM KCl, pH 7.5) was added, and the immobilization reaction was incubated at 37 °C for 2 h. The hairpins H1 and H2 were heated to 90 °C for 5 min followed by slowly decreasing the temperature to 25 °C at a rate of 0.1 °C/s in order to ensure proper folding. Folded hairpins were incubated with different concentrations of C1 at 37 °C for 1.5 h. Each well was washed three times with 200 μL wash buffer before adding the CHA reaction. The CHA products then bound to the Biotin-antisense following incubation at 37 °C for another 1.5 h. Unreacted oligonucleotides were removed by gently washing the plates three times with 200 μL wash buffer, and the HCR reaction was initiated by adding 100 μL of 100 μM H3 and H4 (the HCR system). The plate was kept at 25 °C overnight before measurement or further reaction. 

### 3.3. G-quadruplex with Hemin Catalysis System

A G-quadruplex that could hybridize to the single-stranded tails of H3 and H4 was added to wells where HCR was carried out. After adding 100 μL 100 μM of a previously utilized G-quartet, EAD2 (G1) [20] and incubating for 1 h, the plate was washed 200 μL wash buffer for three times and then 100 μL HPA buffer (2 mM HPA; 0.003% H_2_O_2_; 30 nM Hemin; 0.05% Triton; 200 mM Tris, pH 8.5; 200 mM NaCl; 200 mM KCl) was added. The plate was then transferred to a TECAN Safire plate reader for fluorescence measurements with an excitation wavelength of 310 nm and emission wavelength of 410 nm.

## 4. Conclusions

In conclusion, we successfully built a multi-amplifier that utilized three different nucleic acid components: hairpins that executed CHA, hairpins that executed HCR, and G-quadruplex peroxidases. The primary achievement is that nucleic acid circuits can be readily mixed and matched due to the extraordinary programmability provided by base-pairing interactions. However, these results also demonstrated some of the current limitations of nucleic acid conformational transduction as an amplifier mechanism. Because of either false triggering due to the presence of lesions in the hairpins or because of thermal equilibration of the hairpin structures, the background for each layer was relatively high, and the overall amplification seen was relatively low (100-fold comparable to no amplification). While a low limit of detection could be achieved, quantitation proved difficult. 

The chief advantage of this analytical method is its simplicity and compatibility with surface immobilization. Unlike polymerase-based reactions, which must generally be constructed from multiple components in order to avoid the degradation of reagents or the production of false amplicons, the nucleic acid hairpins are immediately ready for use and must merely be added to an existing sample or surface. Coupled CHA-HCR circuitry can therefore potentially be adapted for the sensitive ‘yes/no’ detection of nucleic acid analytes captured from lysates in point-of-care settings. Such circuitry may also prove to be an interesting intermediate for the ligand-dependent immobilization of nucleic acid-enzyme conjugates, providing basal amplification for ELISA-like assays.

## Supplementary Materials

Supplementary materials can be accessed at: http://www.mdpi.com/1420-3049/17/11/13211/s1.
