# Supplementary material for: Coupling Two Different Nucleic Acid Circuits in an Enzyme-Free Amplifier
Source: Molecules. 2012 Nov 6;17(11):13211–20. doi: 10.3390/molecules171113211 (PMC6268946; doi:10.3390/molecules171113211)

Supporting Information

1. The sequence

Table S1. The sequence used in this work.

Name	*Sequence*	*5'mod*	*3'mod*
**H1**	GTCAGTGA_GCTAGGTT_AGATGTCG_CCATGTGTAGA _CGACATCT_AACCTAGC_CCTTGTCA		
**H2**	AGATGTCG_TCTACACATGG_CGACATCT_AACCTAGC _CCATGTGTAGA_AAGGAGTGTGTGTGCGTGC		
**C1**	CGACATCT_AACCTAGC_TCACTGAC		
**RepF**	CGA_GTGCTCTA_TGACAAGG_GCTAGGTT	FAM	
**RepQ**	C_CCTTGTCA_TAGAGCAC_TCG		IBFQ
**In**	CCATGTGTAGA_AAGGAGTGTGTGTGCGTGC		
**H1-FAM**	GTCAGTGA_GCTAGGTT_AGATGTCG_CCATGTGTAGA _CGACATCT_AACCTAGC_CCTTGTCA	FAM	
**H2-FAM**	AGATGTCG_TCTACACATGG_CGACATCT_AACCTAGC _CCATGTGTAGA_AAGGAGTGTGTGTGCGTGC	FAM	
**H3**	AGGAGTGTGAGTGCGTGCGAAAAGGCACGCACTCAC ACTCCTTTCTACCTTTTTTTTTTTC		
**H4**	CCTTTTCGCACGCACACTCACTCCTGTAGAAAGGAGT GTGAGTGCGTGCTTTTTTTTTTTC		
**H3-FAM**	AGGAGTGTGAGTGCGTGCGAAAAGGCACGCACTCAC ACTCCTTTCTACCTT	FAM	
**H4-FAM**	CCTTTTCGCACGCACACTCACTCCTGTAGAAAGGAGT GTGAGTGCGTGC	FAM	
**G1**	CTGGGAGGGAGGGAGGGA_AAAAAAAAAAAG		
**Bio-Antisense**	TGACAAGG_GCTAGGTT	Biotin	
**FAM-AC**	AACCTAGC_CCTTGTCA	FAM	

2. The kinetics of CHA

Figure S1. The CHA system's kinetic characterization. (**a**) Different concentrations of Catalyst and Hairpins from 1 to 5 were: 5 nM, 1 nM, 0.5 nM, 0 nM, 0 nM. Except for curve 5 which has no hairpins added, the hairpin of H1 and H2's concentration were: 50 nM and 400 nM, respectively. All the reactions were involved with 50 nM reporter; (**b**) The electrophoresis result of HCR with different concentrations and reaction time: From left to right (1 to 8) the reaction time for HCR: overnight (without In), 30 min, 1 h, 2 h, 3 h, 4 h, 5 h and 6 h with the H1 and H2 consistently 200 nM respectively except for lane 1.

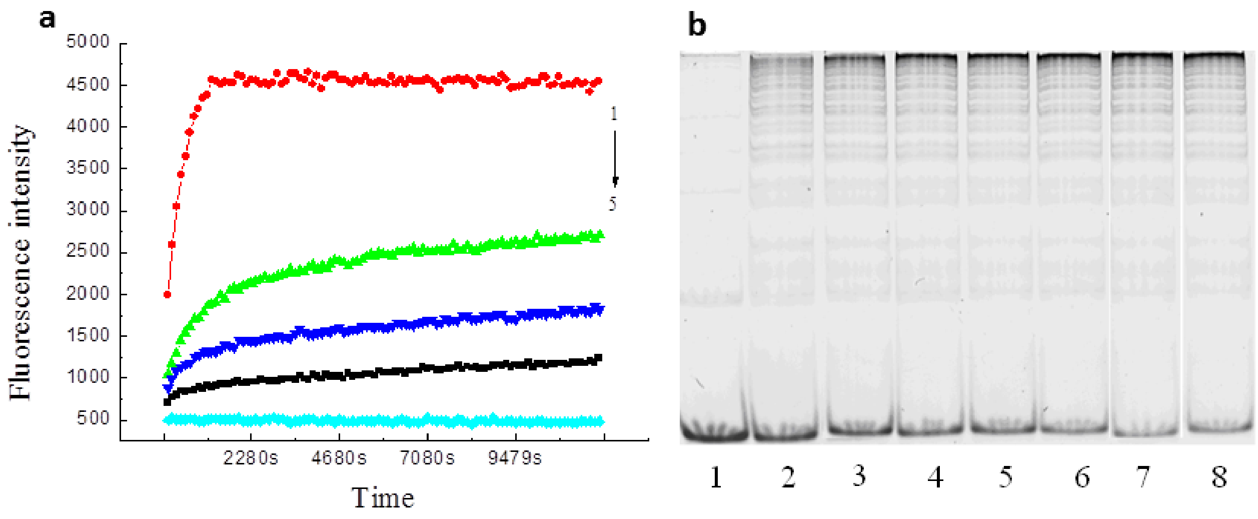

Figure S2. Concentration dependence of G1's homogenous reaction with HPA, with pH 8.5, 1 mM H_2O_2, 20 mM HPA, 5 nM hemin at 37 °C.

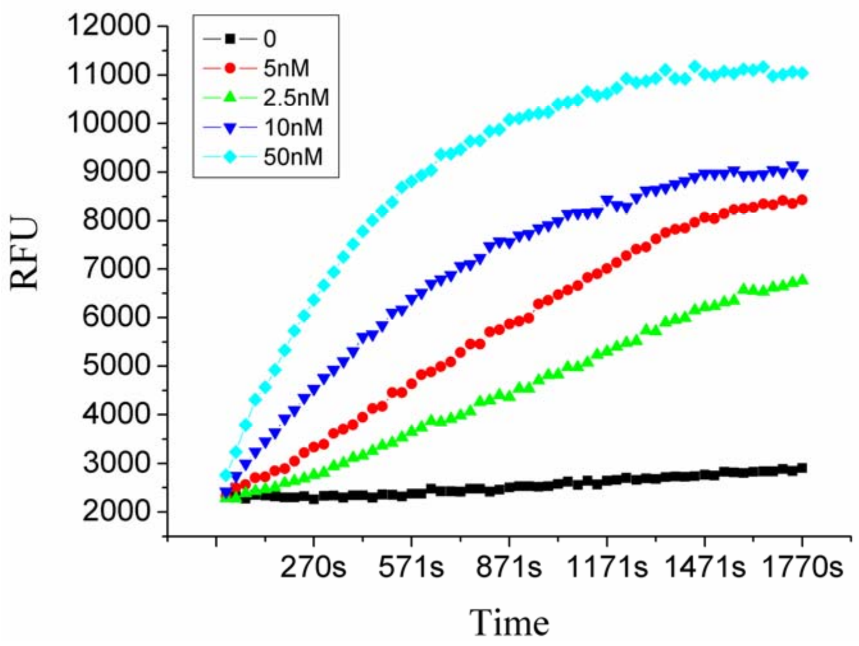

Figure S3. Fluorescent kinetic measurement with CHA-HCR-DNAzyme amplification with different concentrations of C1.

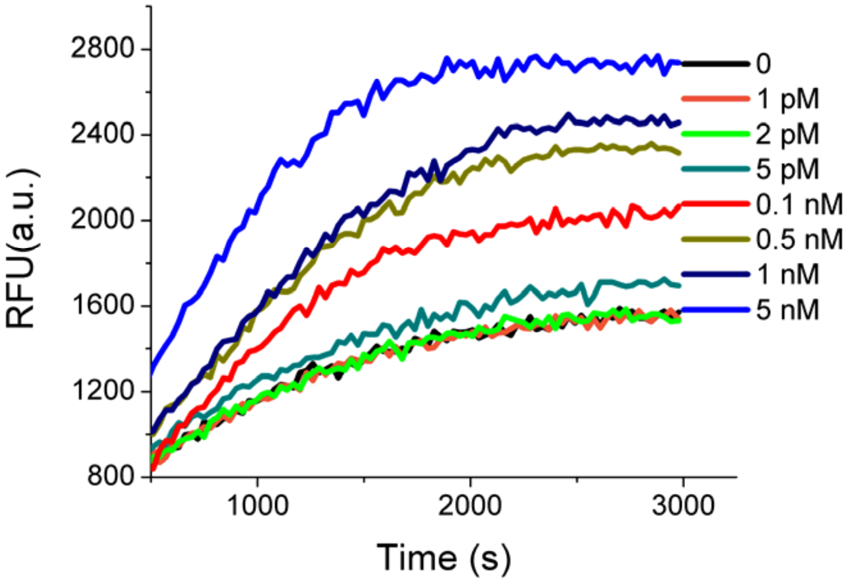

Supplement: Supplementary file 1 [file molecules-17-13211-s001.pdf]
